# Secondary mitral regurgitation—Insights from microRNA assessment

**DOI:** 10.1111/eci.13381

**Published:** 2020-09-10

**Authors:** Georg Spinka, Philipp E. Bartko, Noemi Pavo, Claudia Freitag, Katrin Zlabinger, Suriya Prausmüller, Henrike Arfsten, Gregor Heitzinger, Julia Mascherbauer, Christian Hengstenberg, Mariann Gyöngyösi, Martin Hülsmann, Georg Goliasch

**Affiliations:** ^1^ Department of Internal Medicine II Medical University of Vienna Vienna Austria

**Keywords:** fibrosis, heart failure with reduced ejection fraction, hypertrophy, microRNA, miR‐133a, secondary mitral regurgitation

## Abstract

**Background:**

While secondary mitral regurgitation (sMR) is associated with adverse outcome in heart failure with reduced ejection fraction (HFrEF), key pathophysiologic mechanisms remain poorly understood and might be elucidated by microRNAs (miRNA/miR), that were recently related to cardiac remodelling. This study sought to assess (i) the differences of miRNA profiles in patients with severe sMR compared to matched disease controls, (ii) the correlation between circulating miRNAs and surrogates of sMR severity as well as (iii) the prognostic implications of miRNA levels in severe sMR.

**Materials and methods:**

Sixty‐six HFrEF patients were included, of these 44 patients with severe sMR 2:1 matched to HFrEF controls with no/mild sMR. A comprehensive set of miRNAs (miR‐21, miR‐29a, miR‐122, miR‐132, miR‐133a, miR‐let7i) were measured and correlated to echocardiographic sMR severity.

**Results:**

miRNA patterns differed distinctly between patients with severe sMR and HFrEF controls (*P* < .05). Among the panel of assessed miRNAs, miR‐133a correlated most strongly with surrogates of sMR severity (*r* = −0.41, *P* = .001 with sMR vena contracta width). Interestingly, elevated levels of miR‐133 were associated with an increased risk for cardiovascular death and/or HF hospitalizations with and adjusted HR of 1.85 (95% CI 1.24‐2.76, *P* = .003).

**Conclusions:**

This study unveils distinct pathophysiologic maladaptions at a cellular level in patients with severe sMR compared to no/mild sMR by showing significant differences in miRNA profiles and correlations with sMR severity, supporting the concept that sMR drives cardiac remodelling in heart failure. Moreover, the increased risk for adverse outcome in HFrEF patients with severe sMR conveyed by miR‐133a might indicate irreversible myocardial damage.

## 1 INTRODUCTION

Significant secondary mitral regurgitation (sMR) affects up to 30% of patients with heart failure with reduced ejection fraction (HFrEF) and is associated with poor prognosis and impaired quality of life.^1^ The underlying mechanisms for the development of sMR involve structural alterations of myocardial geometry with subsequent impairment of valvular function. In patients with HFrEF, the most common causes of sMR include left ventricular (LV) dilatation with consecutive displacement of the papillary muscles causing tethering of the mitral leaflets, thereby inducing insufficient coaptation and closure of the mitral valve.^2^, ^3^ Intensive research in recent years focusing on the prognostic impact of quantitatively assessed sMR demonstrated the detrimental effects of the mitral regurgitant load on the failing heart with involvement of fibrotic and hypertrophic remodelling.^4^, ^5^, ^6^ Contemporary data regarding transcatheter mitral valve repair techniques further support this notion by showing clinical improvements through reduction of the regurgitant load^7^; therefore, knowledge regarding macroscopic mechanisms of sMR is beginning to consolidate.

In contrast, pathophysiologic changes on a cellular, biochemical or genetic level associated with sMR are poorly understood. The recent discovery of microRNAs (miRNA/miR), a class of small noncoding RNAs involved in post‐transcriptional regulation of messenger RNAs (mRNA), has inaugurated a new era in pathophysiologic understanding of cardiovascular diseases including heart failure (HF) and its sequelae. Notably, changes in miRNA‐expression generally precede structural alterations, thereby acting as biomarkers possibly able to predict an eventual phenotype of the disease. While specific miRNAs currently savour awareness in context of HF (miR‐21,‐29a,‐122,‐132,‐133a,‐let7i),^8^, ^9^, ^10^ their relationship with sMR remain obscured. This study therefore sought to assess (i) the differences in circulating miRNA profiles in advanced heart failure patients with severe sMR compared to patients with no/mild sMR serving as disease control, (ii) the correlation between quantified surrogates of sMR severity and circulating miRNAs, and (iii) the prognostic impact of miRNA levels on morbidity and mortality in patients with HFrEF and sMR.

## 2 METHODS

### 2.1 Study population and study endpoints

Consecutive HFrEF patients with severe sMR at the heart failure outpatient clinic of the Vienna General Hospital, a university‐affiliated tertiary care centre, were enrolled in this pilot study. We further included HFrEF patients showing no or only mild (no/mild) sMR, serving as disease controls. At study enrolment, we recorded medical history including guideline‐recommended cardiovascular risk factors,^11^ current medication and electrocardiogram recording of all patients. Venous blood samples were collected according to the local laboratory's standard procedure in order to analyse routine laboratory parameters. According to the current heart failure guidelines, HFrEF was defined as a history of heart failure signs and symptoms and a history of LV ejection fraction below 40%.^12^ We then matched patients with HFrEF and severe sMR to patients with HFrEF and no/mild sMR in a 2:1 ratio frequency matched on gender. Patients with primary mitral regurgitation as well as patients with more than mild aortic or mitral stenosis were excluded. The composite of cardiovascular death and/or the first hospitalization for heart failure was chosen as the primary study endpoint. Mortality was assessed via inquiry of the Austrian Death Registry. Further information on hospitalizations for heart failure was retrieved from the centralized patient management system of Vienna (AKIM‐AKH‐Informationsmanagement), which allows a comprehensive overview of patient data from the Vienna General Hospital as well as from every hospital of the Vienna Hospital Association (KAV). The study was approved by the Ethics Committee of the Medical University of Vienna. Reporting of the study conforms to broad EQUATOR guidelines.^13^

### 2.2 Echocardiographic assessment

Commercially available equipment (Vivid5, Vivid7 GE Healthcare) was used to perform standard comprehensive echocardiograms at index time. Cardiac chamber sizes were assessed using diameters in standard four‐ and two‐chamber views, and LV ejection fraction was calculated using the biplane Simpson method. Right ventricular function was assessed semi‐quantitatively by experienced echocardiographers using multiple windows and graded as mild, mild‐to‐moderate, moderate, moderate‐to‐severe and severe according to the current guidelines.^14^ We semi‐quantitatively graded secondary mitral regurgitation using an integrated approach comprising the width of the proximal regurgitant jet (sMR vcw) and the regurgitant jet area (sMR jet area) as previously described.^15^ Valvular regurgitation and stenosis were assessed according to the current guidelines.^16^ Systolic pulmonary artery pressures were calculated by adding the estimated right atrial pressure to the peak tricuspid regurgitation systolic gradient.

### 2.3 Assessment of circulating microRNA

We selected a total of 6 miRNAs previously studied in the context of heart failure for this investigation: miR‐21,‐29a,‐122,‐132,‐133a,‐let7i. In order to assess circulating miRNA‐concentrations, venous blood samples were collected from a peripheral vein, processed to eliminate all blood cells and stored at −80°C. Total RNA including small RNA was extracted using the miRNeasy Mini Kit (Qiagen, Germany) on a Qiacube according to the manufacturer's instruction. RNA quantities were assessed on a NanoDrop 1000 (Thermo Fisher, Germany). All miRNAs are polyadenylated by poly(A) polymerase. Tailed miRNAs are then reverse transcribed using an oligo dT priming strategy. The resulting cDNA is amplified using specific primers (miR‐21: CGTAGCTAGCTTATCAGACTG; miR‐29a: CGGACCTAGCACCATCTGAA; miR‐122: CGCAGTGGAGTGTGACAATG; miR‐132: GTCACTAACAGTCTACAGCC; miR‐133a: CGTAGTTGGTCCCCTTCACCA; let‐7i: GCAGTGAGGTAGTAGGTTGT) using the miScript II RT Kit (Qiagen) according to the manufacturer's instructions. The PCR reaction is monitored in real‐time using the miScript SYBR green PCR Kit (Qiagen) on an Applied Biosystems QuantStudio 5 system (Thermo Fisher). A standard curve was prepared and run to correct values for qPCR efficiency. Expression quantities were calculated with the Thermo Fisher software and expressed relative to Ce‐miR‐39 serving as reference miRNA, thus presented without unit.

### 2.4 Statistical analysis

Discrete data were presented as count and percentage and analysed using the chi‐square test. Continuous data were presented as mean and standard deviation, tested for normal distribution using the Shapiro‐Wilk test and compared as appropriate using independent t test or Kruskal‐Wallis test. miRNAs were log‐transformed for further analysis. Tukey boxplots were used to display the miRNA profiles according to sMR severity. The correlation between miRNA levels and the metrics of mitral regurgitation was assessed calculating Spearman's rho correlation coefficient and displayed using scatter plots. Cox proportional hazard regression analysis was applied to assess the effect of miRNA levels on the outcome of the primary endpoint. Results are displayed as hazard ratio (HR) and 95% confidence intervals (CI). To account for potential confounding effects, we formed a clinical confounder cluster encompassing age, aetiology of HF, kidney function and diuretic therapy. Receiver operating characteristic curve (ROC) analysis was used to assess the discriminatory power of the different miRNAs. To assess time‐dependent discriminative power of miR‐133a levels in patients with severe sMR, we applied the Kaplan‐Meier analysis (log‐rank test). Two‐sided *P*‐values <.05 were considered statistically significant.

## 3 RESULTS

### 3.1 Baseline characteristics

A total of 66 patients with HFrEF were included in this study, median age was 62 ± 15 years, and 51 patients (77%) were male. Forty‐four patients had severe sMR, while the 22 matched control patients showed no/mild sMR. LV function (LVF) was severely reduced in 57 patients (86%), an ischaemic aetiology of HF was present in 21 patients (32%). Fifty‐four per cent of patients (n = 36) were in New York Heart Association (NYHA) functional class II and 21% (n = 14) were in NYHA functional class III. Sixty‐three patients (95%) received Renin‐angiotensin‐aldosterone‐system inhibitors (RASi) up‐titrated to a median dose of 100% of the maximal guideline‐recommended dosages, 65 patients (98%) were treated with beta‐blockers up‐titrated to a median dose of 100% of the maximal guideline‐recommended dosages. Patients were treated with mineralocorticoid receptor‐antagonists in 79% (n = 52), and 28 patients (42%) were under diuretic therapy. Detailed baseline characteristics according to sMR severity are shown in Table S1.

### 3.2 Association of echocardiographic parameters and microRNA levels according to sMR severity

**Figure 1:**
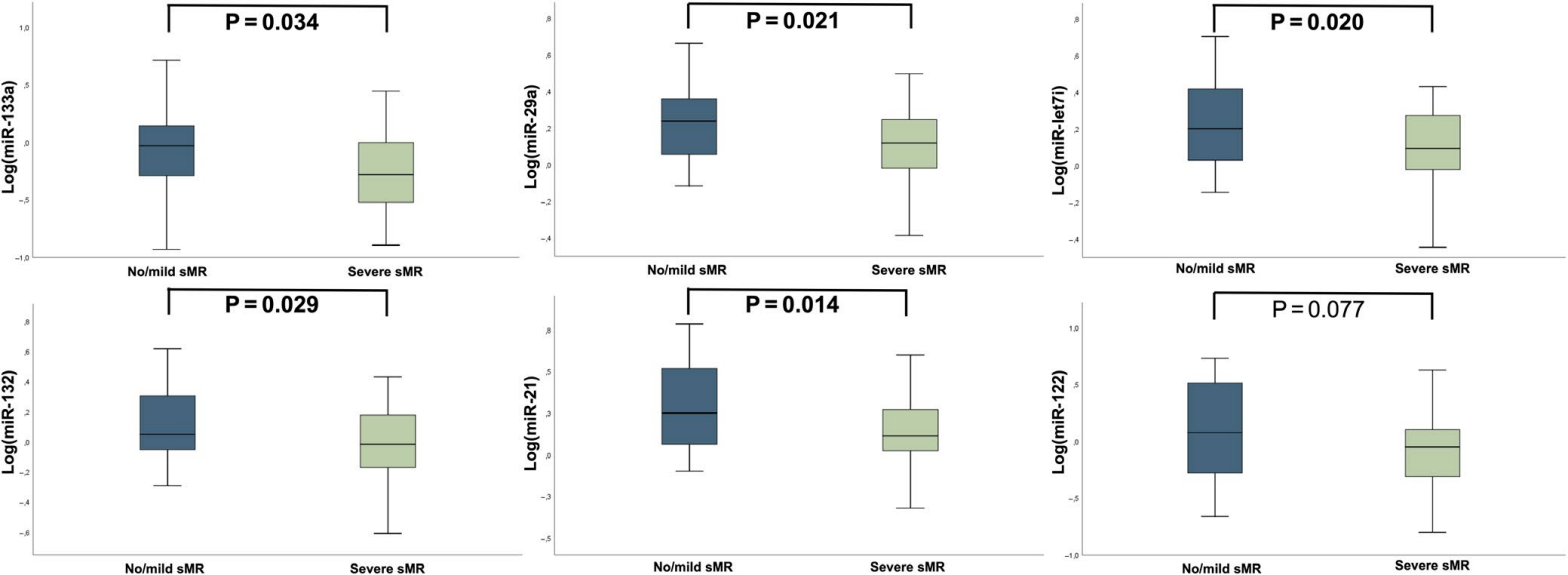
miRNA profiles in HFrEF patients with either severe sMR or no/mild sMR (matched controls). MicroRNA profiles are displayed as Tukey boxplots, comparisons between patients with severe sMR and matched controls were analysed by an independent t test

We observed a significant difference between patients with severe sMR and matched controls regarding the log‐transformed levels of miR‐133a [severe sMR −0.25 ± 0.32 vs no/mild sMR −0.05 ± 0.41, *P* = .034], miR‐let7i [severe sMR 0.09 ± 0.22 vs 0.23 ± 0.24, *P* = .020], miR‐29a [severe sMR 0.11 ± 0.21 vs no/mild sMR 0.23 ± 0.2, *P* = .021], miR‐21 [severe sMR 0.13 ± 0.25 vs no/mild sMR 0.30 ± 0.28, *P* = .014] and miR‐132 [severe sMR −0.03 ± 0.26 vs no/mild sMR 0.12 ± 0.24, *P* = .029]. No significant difference between the two groups was observed for miR‐122. Detailed miRNA profiles according to sMR severity are illustrated in Figure 1.

**Figure 2:**
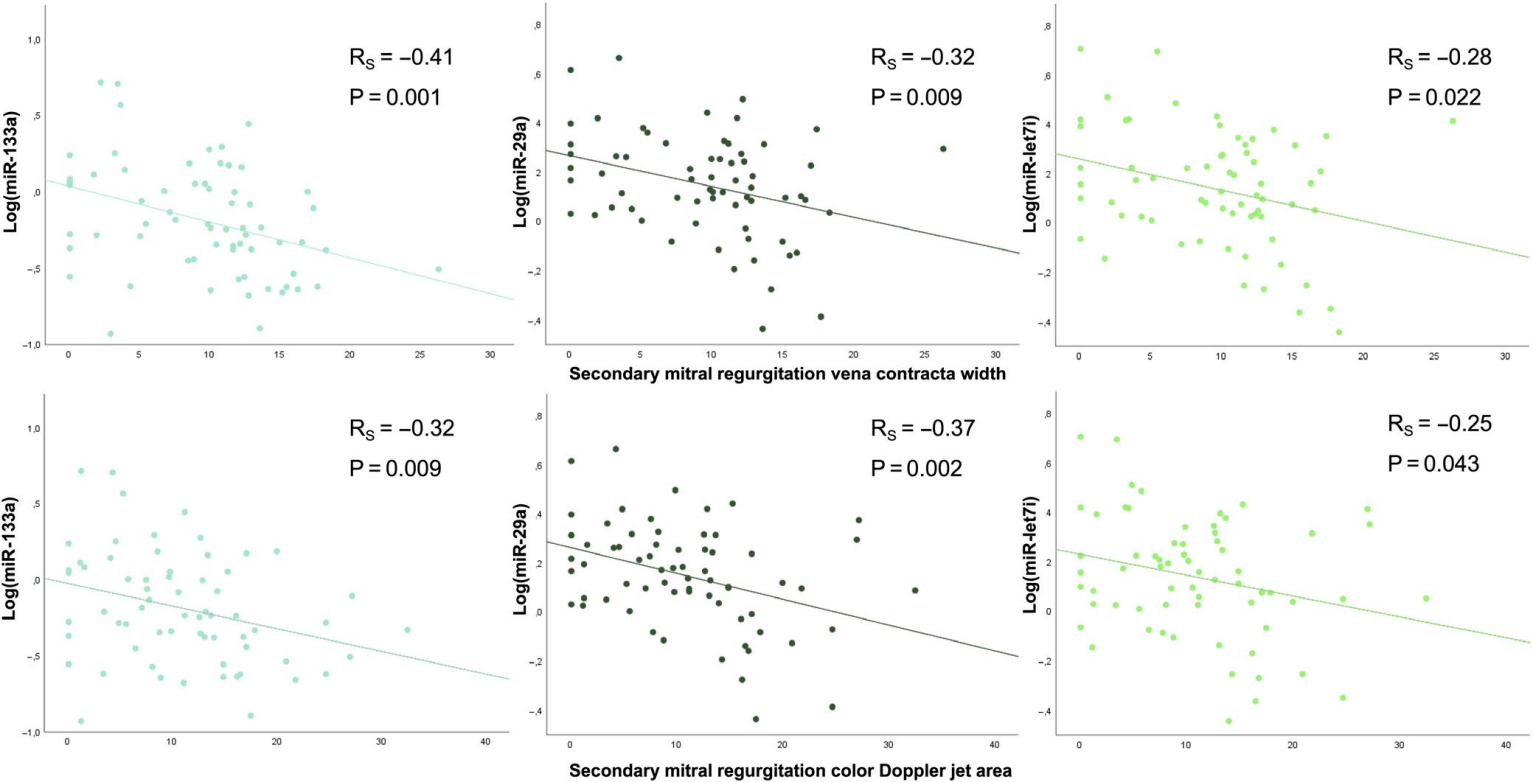
Scatter plot displaying the association between quantified surrogates of sMR (ie sMR vena contracta width and sMR regurgitant jet area) and microRNA levels in patients with HFrEF and severe sMR or no/mild sMR (matched controls). The correlation between the aforementioned variables was assessed using Spearman's rho correlation analysis

Moreover, we observed a significant correlation between sMR vena contracta width and levels of various miRNAs (miR‐133a: *r* = −0.41, *P* = .001; miR‐let7i: *r* = −0.28, *P* = .022; miR‐29a: *r* = −0.32, *P* = .009; miR‐132: *r* = −0.27, *P* = .028) as well as sMR regurgitant jet area and levels of various miRNAs (miR‐133a: *r* = −0.32, *P* = .009; miR‐29a: *r* = −0.37, *P* = .002; miR‐let7i: *r* = −0.25, *P* = .043). Interestingly, we did not observe significant correlations of miRNAs with LV end‐diastolic volumes (for all *P* > .267), NT‐proBNP (for all *P* > .293) or the dosage of diuretic therapy (for all *P* > .361). The relationship between miRNA levels and sMR vcw as well as sMR jet area is depicted in Figure 2. Detailed echocardiographic characteristics and levels of all aforementioned miRNAs according to sMR severity are displayed in Table 1.

**Table 1:** Echocardiographic characteristics and levels of miRNA according to severity of sMR

	Total study population (n = 66)	No/mild sMR (n = 22)	Severe sMR (n = 44)	*P*‐Value (No/mild sMR vs severe sMR)
Echocardiographic characteristics				
Left ventricular end‐diastolic diameter, mm	61 ± 10	58 ± 10	63 ± 10	**.041**
Left ventricular end‐diastolic volume, mL	210 ± 82	190 ± 74	220 ± 84	.160
Left ventricular function				.447
Moderately reduced (LVEF 30%‐40%), n (%)	9 (14)	4 (18)	5 (11)	
Severely reduced (LVEF < 30%), n (%)	57 (86)	18 (82)	39 (89)	
Left atrial volume, mL	86 ± 35	66 ± 24	96 ± 35	**.001**
Right ventricular end‐diastolic diameter, mm	39 ± 7	38 ± 8	39 ± 7	.530
Right atrial diameter, mm	63 ± 10	59 ± 9	65 ± 10	**.023**
MR vena contracta width, mm	9.6 ± 5.6	3.2 ± 2.9	12.8 ± 3.4	**<.001**
MR jet area, cm^2^	10.9 ± 7.4	3.2 ± 2.8	14.8 ± 5.9	**<.001**
Systolic pulmonary artery pressure, mm Hg	51 ± 14	46 ± 11	53 ± 15	.160
miRNA				
miR‐21	0.19 ± 0.27	0.30 ± 0.28	0.13 ± 0.25	**.014**
miR‐29a	0.15 ± 0.21	0.23 ± 0.2	0.11 ± 0.21	**.021**
miR‐122	−0.02 ± 0.37	0.1 ± 0.4	−0.07 ± 0.34	.077
miR‐132	0.02 ± 0.26	0.12 ± 0.24	−0.03 ± 0.26	**.029**
miR‐133a	−0.19 ± 0.36	−0.05 ± 0.41	−0.25 ± 0.32	**.034**
miR‐let7i	0.14 ± 0.24	0.23 ± 0.24	0.09 ± 0.22	**.020**

Note

Bold values indicate statistical significance. miRNAs were log‐transformed prior to analysis. MR indicates mitral regurgitation.

### 3.3 MicroRNA levels and outcome in severe sMR

**Figure 3:**
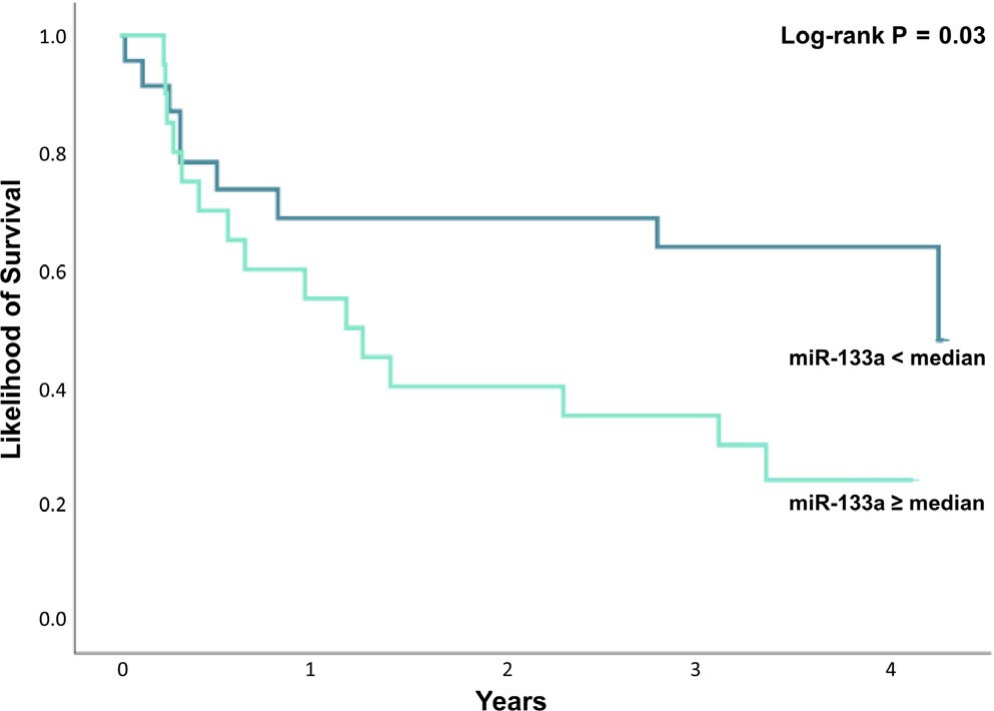
Kaplan‐Meier estimates of the primary outcome comparing patients with severe sMR and miR‐133a levels below the median to patients with severe sMR and miR‐133a levels above the median (log‐rank *P* = .03)

During a median follow‐up of 28 ± 19 months, the primary endpoint defined as cardiovascular death and/or hospitalization for heart failure was reached in 33 patients with severe sMR. Levels of miR‐133a were associated with the primary outcome in the crude cox regression analysis with an HR of 1.76 (95% CI 1.24‐2.51, *P* = .002) with a ROC of 0.72. The results remained virtually unchanged after multivariate adjustment for age, aetiology of HF, kidney function and diuretic therapy with an adjusted HR of 1.85 (95% CI 1.24‐3.13, *P* = .004). Detailed results of the univariable and multivariable cox regression analysis of all assessed miRNAs are displayed in Table 2. Furthermore, Kaplan‐Meier analysis revealed a significant increase in the primary outcome for patients with severe sMR and miR‐133a levels above the median compared to patients with severe sMR and miR‐133a levels below the median (Log‐rank *P* = .03) and is displayed in Figure 3.

**Table 2:** Univariable and multivariable cox regression analysis assessing the impact of miRNA levels on the risk of cardiovascular death and/or heart failure hospitalization

	Univariable model					Multivariable model		
	SD	HR	95% CI	*P*‐value	ROC	Adj. HR^a^	95% CI	*P*‐value
miRNA								
miR133a	0.363	1.76	1.24‐2.51	**.002**	0.72	1.97	1.24‐3.13	**.004**
miR21	0.263	1.10	0.78‐1.55	.597	0.55	0.96	0.67‐1.37	.818
miR132	0.267	1.09	0.76‐1.57	.647	0.55	0.99	0.70‐1.43	.994
miRlet7i	0.239	1.13	0.79‐1.61	.501	0.57	1.02	0.69‐1.51	.934
miR122	0.366	0.79	0.56‐1.12	.209	0.58	0.93	0.64‐1.38	.731
miR29a	0.211	1.19	0.83‐1.70	.347	0.57	1.18	0.78‐1.77	.434

Note

Bold values indicate statistical significance.

^a^ Adjusted for age, aetiology of HF, kidney function and diuretic therapy.

## 4 DISCUSSION

This pilot study investigates for the first time pathophysiologic maladaptations in sMR at a cellular level by revealing an association with designated miRNAs. While most of the assessed miRNA profiles significantly differed between patients with severe sMR and the matched controls, the data specifically emphasize miR‐133a as closely related to sMR severity. Moreover, miR‐133a correlates with quantified surrogates of sMR severity and shows strong prognostic value regarding cardiovascular mortality and heart failure hospitalizations in this specific patient population.

### 4.1 Relationship between microRNA profiles in heart failure and secondary mitral regurgitation

MicroRNAs are small noncoding RNAs involved in post‐transcriptional gene regulation.^17^ Primary miRNAs are transcribed by RNA polymerase II and further processed into a ≈19‐25 nucleotide double‐stranded RNA.^18^ The ‘mature’ strain of the miRNA usually targets a mRNA with complementary sequence, thereby achieving various degrees of degradation or translational repression.^19^, ^20^ The pursued interest and increasing understanding of circulating miRNAs has led to the intriguing idea of using them as biomarkers, not least in the context of cardiovascular diseases.^21^, ^22^ In this study, we assessed miRNAs that have previously been associated with the development of fibrosis and hypertrophy in HF, that is miR‐133a,‐29a,‐let7i,‐21,‐132,‐122, in order to analyse their association to structural alterations in the specific context of sMR.

We observed major alterations in this comprehensive panel of miRNAs for patients with sMR compared to their matched controls, supporting the notion that miRNAs contribute to remodelling mechanisms resulting from the hemodynamic burden of the mitral regurgitant load that induces wall stress in the failing ventricle.^23^ Carè and colleagues were able to show that the decreased expression of miR‐133a compared to healthy control subjects was associated with the development of cardiac hypertrophy and fibrosis in mouse models,^24^ while increased levels of circulating miR‐133a were observed in myocardial infarction patients and interpreted as marker for cardiomyocyte death.^25^ Similarly, recent studies support miR‐let7i as antihypertrophic agent.^26^, ^27^ The decreased levels of miR‐133a and miR‐let7i in patients with severe sMR compared to matched controls as seen in our study thus might illustrate the morphologic maladaptation evolving from severe sMR—that is among others progressive remodelling and fibrotic alterations of myocardial structures.^1^ Moreover, miR‐133a and miR‐let7i inversely correlated with surrogates of sMR severity, thus implying the activation of remodelling and hypertrophic compensation with the increasing hemodynamic burden of sMR. In contrast, elevated levels of miR‐29a were associated with enhanced fibrosis and hypertrophy in patients with hypertrophic cardiomyopathy (HCM).^21^ However, the authors mention themselves that overexpression of miR‐29a appears to be specific to HCM, as they could not reproduce these results for a valvular‐driven hypertrophy. In accordance, levels of miR‐29a were significantly decreased in patients with severe sMR in our study and inversely correlated with surrogates of sMR severity. Studies report miR‐21 to be overexpressed primarily in fibroblasts of the failing heart and to be involved in multiple key signalling pathways.^28^, ^29^ In our study, miR‐21 possibly indicates a difference between fibrotic remodelling in heart failure with or without significant mitral regurgitation. Reported to exert antiapoptotic effects,^30^ the reduced expression of miR‐132 in this study conceivably demonstrates further detrimental effects of sMR in HFrEF.

Studies have been eager to elucidate the pathophysiologic processes ensuing from sMR and revealed the involvement of hypertrophy and fibrosis in the course of the disease on a macroscopic level.^5^, ^6^ The present data unveil distinct pathophysiologic alterations on a cellular level in patients with HFrEF and severe sMR by showing a significant difference in miRNA profiles compared to disease controls. LVF as well as levels of N‐terminal‐pro‐B‐type natriuretic peptide were similar between the two groups; therefore, a difference in heart failure severity appears unlikely. Interestingly, we did not observe a direct correlation between miRNAs and NT‐proBNP as well as LV size. While the association of NT‐proBNP and LV dilatation with sMR is well established,^1^, ^15^ the results of different studies relating miRNAs to NT‐proBNP were inconsistent.^31^ This potentially implies that miRNAs indicate slightly different remodelling mechanisms than currently used biomarkers and imaging parameters. In the light of emerging transcatheter therapies for the mitral valve where patient selection is crucial,^7^ miRNAs might have the potential to further differentiate between patients with valvular‐driven heart failure^32^ and patients with HFrEF where sMR is a mere bystander. In light of the above‐mentioned studies relating the assessed miRNAs to cardiac remodelling, the diverging miRNA profiles as seen in our study support the concept that sMR leads to the development of fibrosis and hypertrophy in heart failure. However, whether fibrosis and hypertrophy are part of morphologic remodelling mechanisms in order to compensate the regurgitant load in earlier stages of severe mitral regurgitation or on the contrary contribute to the progression of the lesion through malignant remodelling remains to be demonstrated. Since they have been associated with cardiac remodelling in heart failure, dilated cardiomyopathy and following acute myocardial infarction, miR‐1, miR‐34a and miR‐208b might provide important insights in the context of morphologic maladaptation in patients with sMR and are therefore potential targets for further research.^33^, ^34^, ^35^ Moreover, transforming growth factor (TGF)‐β1 has been reported to promote fibrosis as well as leaflet thickening thereby driving ischaemic MR.^36^ Likewise, TGF‐activated kinase (TAK)‐1 has been associated with cardiac hypertrophy and fibrosis following pressure overload.^37^ Since these findings relate to the hypothesized mechanism of valvular‐driven heart failure,^32^ further studies establishing a relation between miRNAs and the aforementioned cellular signalling pathways are warranted.

### 4.2 MicroRNAs and outcome in severe secondary mitral regurgitation

While an increase of sMR severity was associated with decreased levels of miRNA in the present study, we observed with interest that the increased expression of circulating miR‐133a was associated with a roughly 1.8‐fold risk of cardiovascular death and/or heart failure hospitalizations. Furthermore, including solely HFrEF patients with severe sMR in the Kaplan‐Meier analysis, we observed an increased risk of the primary outcome in patients with severe sMR and miR‐133a levels above the median. The downregulation of miR‐133a, which is associated with severe sMR in our study, might display the involvement in physiologic and protective remodelling mechanisms occurring over the natural course of sMR. Notwithstanding, it is the overexpression of miR‐133a bearing the increased risk of the primary outcome in patients with HFrEF and severe sMR, potentially indicating the general lack of these morphologic mechanisms in individual patients or their loss over time. A recent study showed that high sensitivity cardiac troponin T (hs‐TnT) predicted cardiovascular death and/or hospitalizations for HF in patients undergoing percutaneous mitral valve repair.^38^ As mentioned above, increased levels of miR‐133a were related to cardiomyocyte death in myocardial infarction patients and rose even faster than cardiac troponin T.^22^, ^25^ Thus, while levels of miR‐133a are significantly higher in control patients without sMR, the upregulation of miR‐133a in patients already presenting with severe sMR might indicate the late stages of the disease in which cardiac remodelling has progressed to an irreversible level associated with continuing cardiomyocyte damage. In the light of studies investigating the effect of a reduction of mitral regurgitation, the scientific community came to the realization that patients only profited from the intervention when beneficial reverse remodelling was possible after the reduction of the regurgitant load.^1^, ^7^ Based on these findings, the present data would suggest that high miR‐133a levels in patients with severe sMR indicate irreversible maladaptive remodelling possibly resulting in futile mitral edge‐to‐edge repair therapies; however, further studies are needed to confirm these results.

### 4.3 Limitations

We have to acknowledge several limitations of our study, foremost the small sample size of the compared groups. Furthermore, sMR was not assessed by quantitative parameters (ie EROA and regurgitant volume), which would possibly even better reflect the correlation with different miRNAs in our study. Although NYHA Class was reported in all patients, a more sophisticated functional evaluation using the Kansas City Cardiomyopathy Questionnaire would be desirable to more precisely describe the clinical state of the patients. This study displays the experience of a single tertiary care centre. However, this ensures a homogenous recruitment of the study patients while pursuing a comprehensive clinical routine. Accounting for the dynamic nature of atriovalvular regurgitation, the patients were carefully included in a clinically stable condition from our heart failure outpatient clinic; therefore, we can rule out a decompensated stage of HF at the time of miRNA‐assessment.

## 5 CONCLUSION

This pilot study discovered distinct pathophysiologic changes at a cellular level in patients with severe sMR compared to matched controls with HFrEF by showing a strong association with specific miRNAs previously related to HF, supporting sMR as driving force of cardiac remodelling and adverse outcome in heart failure. Specifically, we observed decreased expression of circulating miR‐133a, miR‐29a, miR‐let7i, miR‐21 and miR‐132 in patients with severe sMR compared to gender‐matched controls. Moreover, miR‐133a, miR‐let7i and miR‐29a correlate with semi‐quantitative measures of sMR severity, highlighting their involvement in remodelling mechanisms that potentially compensate the hemodynamic burden of the regurgitant load in earlier stages of severe mitral regurgitation. Finally, higher levels of miR‐133a are associated with an increased risk for cardiovascular death and/or hospitalizations for heart failure when solely considering patients with HFrEF and severe sMR, possibly indicating irreversible myocardial damage.

## CONFLICT OF INTEREST

The authors declare no conflict of interest.

## AUTHOR CONTRIBUTION

Georg Spinka designed and performed the study, analysed data, wrote the first draft and edited the paper. Philipp E. Bartko performed the study, analysed data, reviewed and edited the paper. Noemi Pavo designed the study, contributed important reagents, collected and analysed data, and reviewed the paper. Claudia Freitag contributed important reagents, collected and analysed data and reviewed the paper. Katrin Zlabinger contributed important reagents, collected and analysed data and reviewed the paper. Suriya Prausmüller analysed data, reviewed and edited the paper. Henrike Arfsten collected and analysed data and reviewed the paper. Gregor Heitzinger collected and analysed data and reviewed the paper. Julia Mascherbauer designed the study, reviewed and edited the paper. Christian Hengstenberg contributed important reagents and reviewed the paper. Mariann Gyöngyösi designed the study, contributed important reagents and reviewed the paper. Martin Hülsmann designed the study, contributed important reagents, reviewed and edited the paper. Georg Goliasch designed and performed the study, analysed data, contributed important reagents, reviewed and edited the paper.

## Supporting information

Table S1
