# Supplementary material for: Secondary mitral regurgitation—Insights from microRNA assessment
Source: Eur J Clin Invest. 2020 Sep 10;51(2):e13381. doi: 10.1111/eci.13381 (PMC7900984; doi:10.1111/eci.13381)
Supplement: Supplementary file 1 — Table S1 [file ECI-51-e13381-s001.docx]

**Tables**

**Table 1.** Baseline characteristics of the total study population and according to severity of secondary mitral regurgitation

|  | Total study population (n=66) | No/mild sMR (n=22) | Severe sMR (n=44) | P-Value (No/mild sMR vs. severe sMR) |
| --- | --- | --- | --- | --- |
| **Baseline characteristics** | | | | |
| Age, years | 62±15 | 58±16 | 64±14 | 0.173 |
| Male sex, n (%) | 51 (77) | 17 (77) | 34 (77) | 1.000 |
| Body-Mass-Index, kg/m² | 26.6±4.9 | 27.6±6 | 26.1±4.3 | 0.337 |
| Ischemic etiology of HF, n (%) | 21 (32) | 8 (36) | 13 (30) | 0.575 |
| Diabetes mellitus, n (%) | 15 (23) | 6 (27) | 9 (20) | 0.533 |
| Hypertension, n (%) | 41 (62) | 16 (73) | 25 (57) | 0.209 |
| Hyperlipidemia, n (%) | 30 (45) | 15 (68) | 15 (34) | **0.009** |
| Atrial fibrillation, n (%) | 25 (38) | 7 (32) | 18 (41) | 0.473 |
| NYHA functional class |  |  |  | 0.179 |
| NYHA functional class II | 36 (54) | 15 (68) | 21 (48) |  |
| NYHA functional class III | 14 (21) | 2 (9) | 12 (27) |  |
| Creatinine, mg/dL | 1.34±0.61 | 1.16±0.36 | 1.43±0.69 | 0.157 |
| NT-proBNP, pg/ml | 4524±6423 | 2810±3213 | 5341±7382 | 0.265 |
| **Devices** | | | | |
| Cardiac resynchronization therapy, n (%) | 10 (15) | 3 (14) | 7 (16) | 0.808 |
| Implantable cardioverter-defibrillator, n (%) | 25 (38) | 8 (36) | 17 (39) | 0.858 |
| Pacemaker, n (% | 6 (9) | 1 (5) | 5 (11) | 0.364 |
| **Medication** | | | | |
| Beta-Blockers, n (%) | 65 (98) | 22 (100%) | 43 (98) | 0.476 |
| Percent of maximal recommended dose, median % | 100 | 100 | 100 | 0.819 |
| RASi, n (%) | 63 (95) | 22 (100%) | 41 (93) | 0.210 |
| Percent of maximal recommended dose, median % | 100 | 100 | 100 | 0.382 |
| MRA, n (%) | 52 (79) | 17 (77) | 35 (80) | 0.831 |
| Percent of maximal recommended dose, median % | 100 | 100 | 100 | 0.566 |
| Furosemide, n (%) | 28 (42) | 11 (50) | 17 (39) | 0.379 |
| Dosage, median % | 40 (15-60) | 20 (10-50) | 40 (20-60) | 0.417 |

Bold values indicate statistical significance.

IQR indicates interquartile range; LVEF, left ventricular ejection fraction; MRA, mineralocorticoidreceptor-antagonist; NT-proBNP, N-terminal-pro-natriuretic peptide; NYHA, New York Heart Association; RASi, Renin-angiotensin-aldosterone-system inhibitors; sMR, secondary mitral regurgitation.
